# Hepatocyte cannabinoid 1 receptor nullification alleviates toxin-induced liver damage via NF-κB signaling

**DOI:** 10.1038/s41419-020-03261-8

**Published:** 2020-12-09

**Authors:** Yoo Kim, Sudeep Gautam, Kanikkai Raja Aseer, Jaekwan Kim, Prabha Chandrasekaran, Caio Henrique Mazucanti, Paritosh Ghosh, Jennifer F. O’Connell, Máire E. Doyle, Ashley Appleton, Elin Lehrmann, Qing-Rong Liu, Josephine M. Egan

**Affiliations:** 1 Laboratory of Clinical Investigation, National Institute on Aging, National Institutes of Health, Baltimore, MD USA; 2 Department of Nutritional Sciences, Oklahoma State University, Stillwater, OK USA; 3 Laboratory of Molecular Biology and Immunology, National Institute on Aging, National Institutes of Health, Baltimore, MD USA; 4 Laboratory of Genetics and Genomics, National Institute on Aging, National Institutes of Health, Baltimore, MD USA

**Keywords:** Autoimmune hepatitis, Acute inflammation

## Abstract

Cannabinoid 1 receptor (CB1R) expression is upregulated in the liver with viral hepatitis, cirrhosis, and both alcoholic and non-alcoholic fatty liver disease (FLD), whereas its expression is muted under usual physiological conditions. Inhibiting CB1R has been shown to be beneficial in preserving hepatic function in FLD but it is unclear if inhibiting CB1R during an inflammatory response to an acute hepatic injury, such as toxin-induced injury, would also be beneficial. We found that intrinsic CB1R in hepatocytes regulated liver inflammation-related gene transcription. We tested if nullification of hepatocyte-specific CB1R (hCNR1^−/−^) in mice protects against concanavalin A (Con A)-induced liver injury. We looked for evidence of liver damage and markers of inflammation in response to Con A by measuring liver enzyme levels and proinflammatory cytokines (e.g., TNF-α, IL-1β, IL-6, IL-17) in serum collected from hCNR1^−/−^ and control mice. We observed a shift to the right in the dose-response curve for liver injury and inflammation in hCNR1^−/−^ mice. We also found less inflammatory cell infiltration and focal necrosis in livers of hCNR1^−/−^ mice compared to controls, resulting from downregulated apoptotic markers. This anti-apoptotic mechanism results from increased activation of nuclear factor kappa B (NF-κB), especially cAMP-dependent cannabinoid signaling and membrane-bound TNF-α, via downregulated TNF-α receptor 2 (TNFR2) transcription levels. Collectively, these findings provide insight into involvement of CB1R in the pathogenesis of acute liver injury.

## Introduction

Inflammatory liver diseases, an increasing health problem worldwide, are caused by diverse agents such as toxins, viruses, various drugs and alcohol ingestion, fatty liver disease (FLD), and immune dysfunction^1^. The first insult leads to hepatocyte injury that is reflected in increased circulating levels of liver enzymes, and, if left unchecked, progressive liver inflammation, hepatocyte apoptosis, necrosis, and fibrosis ultimately results in cirrhosis and liver failure. Concanavalin A (Con A), a Canavalia extract and a plant lectin (carbohydrate-binding protein), has direct hepatotoxic effects^2^. Injecting mice with Con A lead to acute liver injury and the resultant appearance of increased levels of liver enzymes in the circulation as early as 12 h post injection. Liver infiltration with inflammatory lymphocytes, primarily natural killer T (NKT) cells and CD4+ lymphocytes occurs, triggering the secretion of several cytokines, chemokines, and inflammatory mediators^3,4^.

The endocannabinoid system (ECS) consists of two ligands, anandamide and 2-arachidonoylglycerol (2-AG), both of which are lipid mediators, and two endocannabinoid (EC) receptors, cannabinoid 1 (CB1R) and cannabinoid 2 (CB2R): both receptor types are G*i*-protein coupled (GPCRs). The central nervous system (CNS) has the highest expression of CB1R but it is also present in peripheral organs, whereas CB2R is predominantly expressed in immune cells^5,6^. While the expression levels of EC receptors are low in the liver under normal physiological conditions, marked increases in their levels occur upon injury and disease^7,8^. Functional studies indicate opposing effects of CB1R and CB2R in the liver. For example, chronic CB1R activation can result in pro-fibrogenic changes, whereas CB2R activation is anti-fibrogenic^7,9^. Hepatic CB1R, when activated, is reported to play a major detrimental role in the pathogenesis of alcoholic and non-alcoholic FLD^10^. Therefore, pharmacological or genetic inhibition of CB1R, but not CB2R, has been proposed to be a therapeutic strategy to treat liver pathology due to alcohol- and high fat diet-induced FLD^11^.

We, and others, have reported the presence and outlined some physiological actions of CB1R in murine and human hepatocytes, as well as in beta cells in islets of Langerhans^12,13^. A CB1R antagonist, SR141716A, was found to be hepatoprotective with respect to Con A injury^14^, while, in contrast, exogenous and endogenous cannabinoids have been shown to attenuate experimental autoimmune hepatitis^15^. To definitively investigate the involvement of hepatic CB1 receptors in toxin-induced liver damage, we used hepatocyte-specific CB1R-null mice (hCNR1^−/−^) and studied their response to a Con A insult. Our findings show that lack of CB1R exerts a hepatoprotective effect and raises the possibility of using CB1R-specific antagonists or inverse agonists to mitigate against hepatitis during acute toxin injury. We have already reported that human hepatocytes and β-cells contain a specific isoform of CB1R^13^, thus adding a potential for liver specificity of CB1R antagonistic actions in humans.

## Results

### Hepatic Cnr1 gene regulates innate liver damage-related gene profiles

**Fig. 1:**
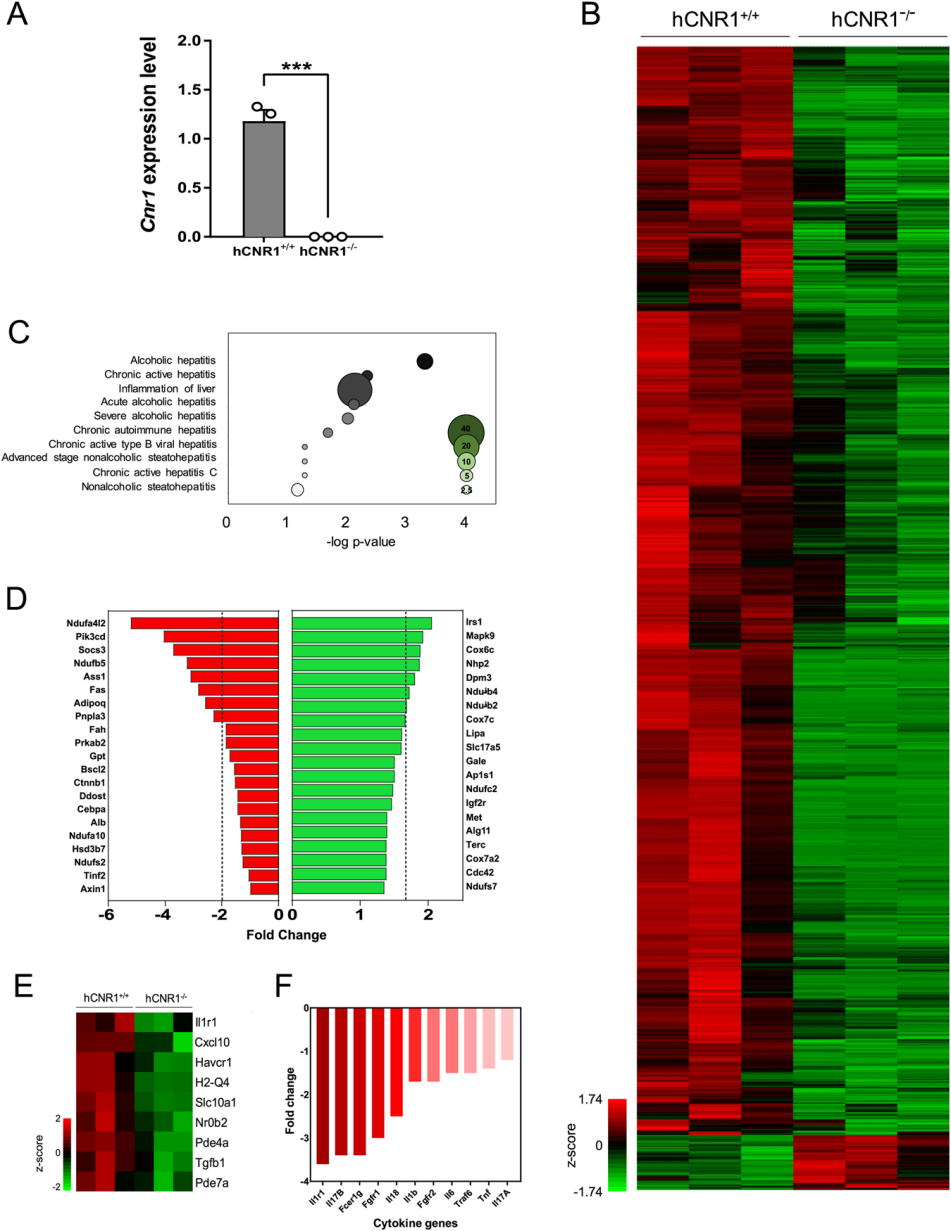
Hepatic Cnr1 regulates liver disease-associated genes. Gene expression using RNA extracted from isolated hepatocytes of hCNR1^+/+^ and hCNR1^−/−^ mice (*n* = 3). **A** CB1R mRNA (*Cnr1*) expression in hepatocytes isolated from hCNR1^+/+^ and hCNR1^−/−^ mice. **B** Heat map of genome-wide expression changes by normalized *z*-score between −1.74 and 1.74. **C** Gene ontology (GO) analysis on liver disease-related genes based on genome-wide expression changes by normalized *z*-score between −1.74 and 1.74 depicted in **B**. Bubble sizes depict number of genes associated with hepatitis category. **D** Fold change of genes that are inflammation associated in hCNR^−/−^ mice compared to hCNR^+/+^ mice. **E** Fold change of genes that are liver-disease associated in hCNR^−/−^ mice compared to hCNR1^+/+^ mice. Heat map showing *z*-score (−2.0 and 2.0) of the nine identified present in the GO pathway associated with liver damage inflammation. **F** Fold change of inflammatory cytokines and growth factors in hCNR^−/−^ mice compared to hCNR1^+/+^ mice.

To investigate the effect of hepatic CB1R on liver damage, we generated hepatocyte-specific *Cnr1* gene knockout mice (Fig. 1A). We profiled global mRNA expression of isolated hepatocytes (devoid of inflammatory cells) from hCNR1^+/+^ and hCNR1^−/−^ mice. Comparison of CB1R null vs. control hepatocyte transcriptomes showed 1423 downregulated and 72 upregulated genes (fold-change >2, *p* < 0.05) (Fig. 1B). We employed analysis of Gene Ontology (GO) based on differentially expressed genes. As expected, we found *Cnr1* gene was highly involved in liver hepatitis and inflammation in hepatocytes (Fig. 1C, D). In hCNR1^−/−^ hepatocytes, 63 out of 95 genes involved in inflammation were downregulated. Further, of 231 genes whose alterations have been implicated in multiple liver diseases such as hepatitis, FLDs, and polycystic liver diseases, 132 genes (57%) were downregulated in hCNR^−/−^ mice (Fig. 1D, E). Also, the genes controlled by inflammatory cytokines such as interleukin 1 receptor 1 (*Il1r1*), interleukin 1β (*Il1b*), interleukin 6 (*Il6*), and tumor necrosis factor (*Tnf*) were substantially downregulated (Fig. 1F). Collectively, ablation of *Cnr1* gene in hepatocytes is characterized by reduced inflammatory and liver injury-associated genes in the resting state, suggesting a role for CB1R, not only under duress, but also under steady-state conditions.

### Deletion of hepatocyte CB1R ameliorates liver damage via anti-apoptotic signaling

**Fig. 2:**
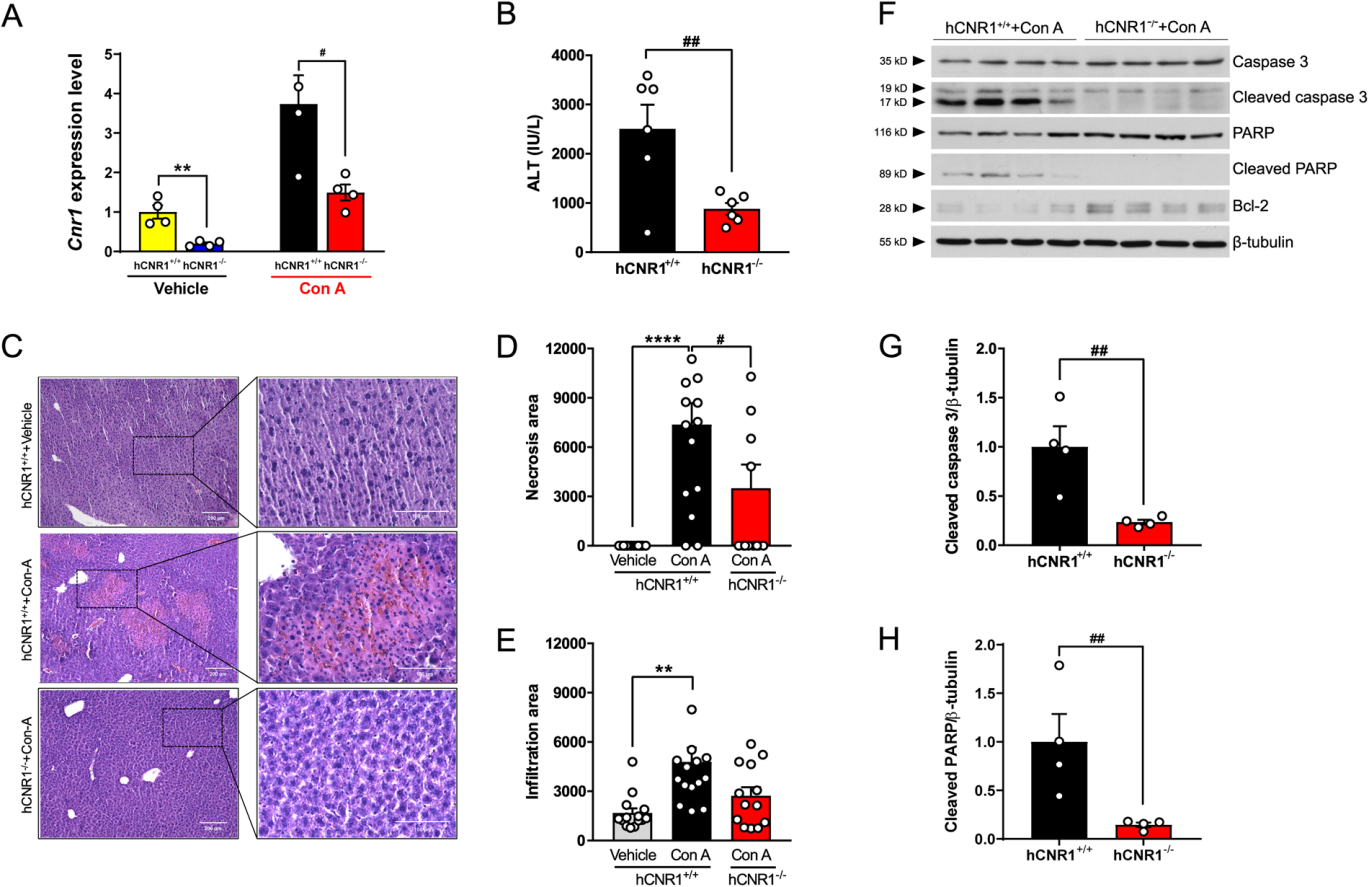
Nullification of hepatic CB1R ameliorates liver damage via anti-apoptotic signaling. **A** *Cnr1* in liver from hCNR1^+/+^ and hCNR1^−/−^ mice with or without Con A (5 mg/kg) injection (*n* = 4–5) for 12 h. **B** Plasma alanine aminotransferase (ALT) levels (IU/L) in Con A-administered hCNR1^+/+^ and hCNR1^−/−^ mice (*n* = 6 per each group). **C**–**E** H&E staining (×10 (left, scale bars, 200 μm) and ×40 (right, scale bars, 100 μm) magnifications) and quantifications of the percentage of the average area of inflammatory cells inflammation (**D**) and hepatocyte necrosis (**E**) after 5 mg/kg of Con A administration for 12 h. **F** Protein expression levels in liver tissue lysates post-injection of 5 mg/kg of Con A administration for 12 h (*n* = 4) and quantification for cleaved caspase 3 (**G**) and poly (ADP-ribose) polymerase (PARP) (**H**). Values were normalized to β-tubulin. Results were expressed as mean ± S.E.M. (*p ≤ 0.05, **p ≤ 0.01, ****p ≤ 0.0001 compared to Control; ^#^*p* ≤ 0.05, ^##^*p* ≤ 0.01, ^###^*p* ≤ 0.001 compared to hCNR1^+/+^+Con A).

To understand if hepatic CB1R has any role during liver injury, we injected hCNR1^+/+^ and hCNR1^−/−^ mice with PBS or Con A. Con A induced an obvious increase of hepatic *Cnr1* mRNA expression levels in the hCNR1^+/+^ mice (Fig. 2a). On the other hand, *Cnr2* mRNA expression levels were not different between the two groups under basal conditions, or with Con A treatment (Fig. S1A, B). In accordance with the in vivo data, freshly isolated hepatocytes from hCNR^−/−^ mice were significantly less sensitive to Con A treatment compared to WT hepatocytes (IC_50_: 133.8 + 7.6 vs. 67.75 + 2.5 µg/mL, respectively; Fig. S2A), indicating their inherent resistance of hepatocytes to Con A treatment.

Next, we determined the effective duration and concentration of intravenous Con A for induction of hepatitis in hCNR1^+/+^ and hCNR1^−/−^ mice (Fig. S2B, C). Con A (5 mg/kg) induced liver injury in hCNR1^+/+^ mice, based on serum alanine aminotransferase (ALT) levels, as early as 3 h after administration with maximum effect at 12 h. In contrast, ALT levels in hCNR1^−/−^ mice were normal until 12 h post administration, then there was a slight elevation that had returned to normal by 24 h (Fig. 2B and S2B). Histological analysis of liver confirmed that hCNR1^−/−^ mice had less inflammatory cell infiltration and liver necrosis (Fig. 2C–E). Notably, scattered focal necrosis was significantly less in hCNR1^−/−^ compared to hCNR1^+/+^ mice (Fig. 2C, E). To investigate whether necrosis was the result of apoptosis, we checked the liver tissue lysates post-injection of Con A (5 mg/kg) for 12 h for apoptosis markers. The levels of cleaved caspase 3, the active form of caspase 3, were significantly higher in hCNR1^+/+^ mice compared to hCNR1^−/−^ mice (Fig. 2F, G). Furthermore, the levels of poly (ADP-ribose) polymerase (PARP), a downstream substrate of caspase 3, was also markedly higher in hCNR1^+/+^ mice (Fig. 2F, H). In contrast, Bcl-2, an anti-apoptotic protein in the intrinsic pathway, was increased in hCNR1^−/−^ compared to hCNR1^+/+^ mice (Fig. 2F), strongly supporting the notion that the deficiency of hepatic CB1R protects against programmed cell death caused by an extrinsic causative factor such as Con A.

### Con A-induced inflammation was suppressed in hCNR1^−/−^ mice

**Fig. 3:**
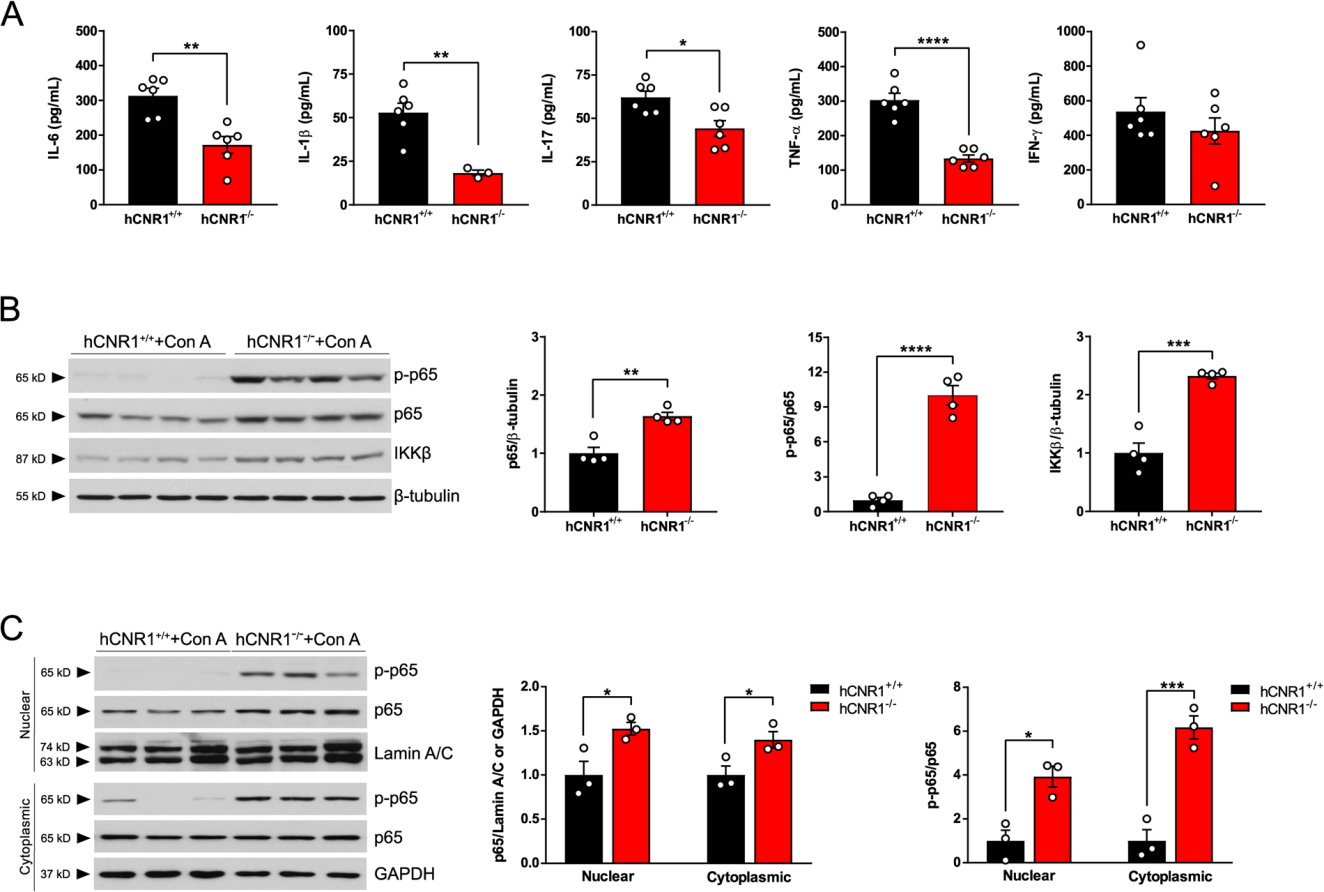
Con A-induced inflammation was suppressed in hCNR1^−/−^ mice. hCNR1^+/+^ and hCNR1^−/−^ mice were injected with Con A (5 mg/kg) for 12 h (*n* = 6). **A** Blood samples were collected post-Con A injection (5 mg/kg) after 12 h period (*n* = 3–6) and measured proinflammatory cytokines, IL-6, IL-1β, IL-17, TNF-α, and IFN-γ. **B** Protein extracts from liver homogenates were immunoblotted and detected by autoradiographic signals. The bands were subjected to densitometric quantifications for protein expression levels of total p65, phospho-p65 (p-p65), and IKKβ (*n* = 4). **C** Total p65 and p-p65 nuclear and cytoplasmic protein amounts in the liver lysates. Nuclear and cytoplasmic fractions were normalized to lamin A/C and GAPDH, respectively. Results were expressed as mean ± S.E.M. (*p ≤ 0.05, **p ≤ 0.01, ***p ≤ 0.001, ****p ≤ 0.0001 compared to hCNR1^+/+^ mice).

Con A-induced liver injury is accompanied by increasing serum levels of proinflammatory cytokines such as interleukin (IL)-6, IL-1β, IL-17, interferon γ (IFN-γ), and tumor necrosis factor α (TNF-α) that lead to induction of hepatic apoptosis^2^. We therefore measured serum levels of these cytokines in Con A-treated mice and we found that all cytokines except for IFN-γ were significantly lower in hCNR1^−/−^ mice (Fig. 3A). The transcription factor nuclear factor kappa B (NF-κB) protects against cytokine-induced apoptosis^16^. Mice lacking p65 subunit of NF-κB suffer from mid-embryonic lethality due to massive liver apoptosis involving TNF-α, suggesting a pivotal role for p65 in protection against TNF-α-induced apoptosis^17^. Thus, we sought to determine the phosphorylation status of the NF-κB p65 subunit since phosphorylation of p65 results in an increase in its transcriptional activity^18^. We found that the levels of phosphorylated p65 were indeed increased in livers of hCNR1^−/−^ mice after Con A injection along with increased total p65 levels, compared to Con A-injected hCNR1^+/+^ mice (Fig. 3B). To confirm this result, we fractionated proteins from cytoplasmic and nuclear cellular compartments, then detected expression levels of phosphorylated and total p65. In keeping with the results from whole lysates, livers from hCNR1^−/−^ mice had significantly higher expression of both phospho- and total p65 in both compartments (Fig. 3C). The kinase responsible for the phosphorylation of p65 is the inhibitor of κB kinase (IKK) β^18^ and we found that the expression levels of IKKβ were significantly upregulated in hCNR1^−/−^ mice (Fig. 3B). IKKβ-deficient hepatocytes are sensitive to Con A-induced liver damage through TNF-α signaling^19^ and mice lacking IKKβ displayed embryonic lethality in mid-gestation^20^, which is identical to the phenotype of p65-deficient mice. Taken together, these data suggest that lack of CB1R is conducive to activation of NF-κB in the liver, which drives anti-apoptotic signaling and consequently protects liver damage because of Con A administration.

### Ablation of hepatic CB1R protects liver damage via cAMP-dependent pathway

**Fig. 4:**
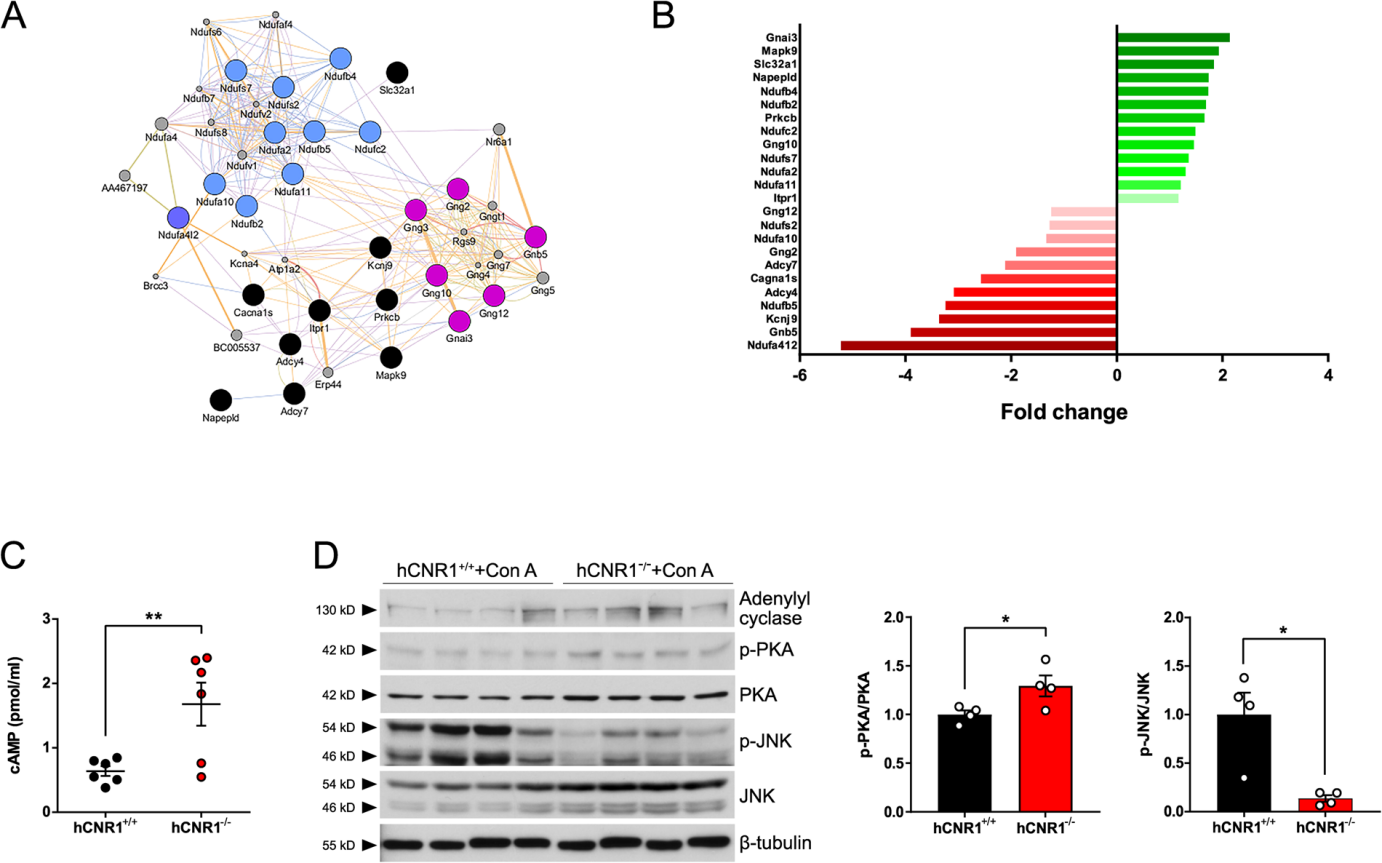
Ablation of hepatic CB1R plays a critical role in cAMP-dependent pathway to rescue liver damage. **A**, **B** Gene network of interactions between the 25 (of 139) significant genes (*p* < 0.05) involved in cannabinoid signaling pathway and their fold change in expression. **C** cAMP levels (pmol/mg) in liver. **D** Liver lysates were prepared and protein expression levels are expressed as a ratio of p-PKA/PKA and ratio of p-JNK/JNK (*p ≤ 0.05, **p ≤ 0.01 compared to hCNR1^+/+^ mice).

CB1R activation is well-documented by us and others to suppress adenylyl cyclase (AC) activity, resulting in reduced intracellular cyclic adenosine monophosphate (cAMP) levels and downstream protein kinase A (PKA) activation^21,22^. To confirm this cellular signal transduction in hepatocytes we analyzed their gene profiles (Fig. 1B). Ninety-four out of 139 genes downstream from EC signaling were downregulated, including the notable adenylate cyclase 4 and 7 (*Adcy4* and *Adcy7*) in hCNR1^+/+^ compared to hCNR1^−/−^ mice (Fig. 4A, B).

Thus, we further investigated whether cAMP is involved in the anti-apoptotic signaling pathway observed in hCNR1^−/−^ mice during Con A-induced liver injury. We first evaluated levels of cAMP in the liver of Con A-treated hCNR1^+/+^ and hCNR1^−/−^ mice: hCNR1^−/−^ mice had increased levels compared to hCNR1^+/+^ mice (Fig. 4C). To elaborate on this finding, we analyzed the protein expression levels of AC and phospho-PKA in liver homogenates. In concurrence with the elevated levels of cAMP, the ratio of phosphorylated PKA and total PKA in hCNR1^−/−^ was significantly higher than in hCNR1^+/+^ mice (Fig. 4D). The expression levels of AC were also significantly higher in hCNR1^−/−^ mice (Fig. 4D). Therefore, we further explored the cAMP signaling and intrinsic apoptotic pathways. CB1R activation and Con A-induced liver injury commonly involve the mitogen-activated protein kinase (MAPK) signaling pathway^23^. Among nodes in MAPK signaling cascades, CB1R signaling leads to activation of extracellular signal-regulated kinase 1/2 (ERK 1/2) and c-Jun N-terminal kinase (JNK), both of which are inhibited by cAMP^24,25^. In contrast to ERK 1/2 phosphorylation, where no difference was observed between the two groups (Fig. S3), phosphorylation of JNK was significantly less in hCNR1^−/−^ compared to hCNR1^+/+^ mice (Fig. 4D).

### Role of membrane-bound TNF-α in Con A-induced liver damage

**Fig. 5:**
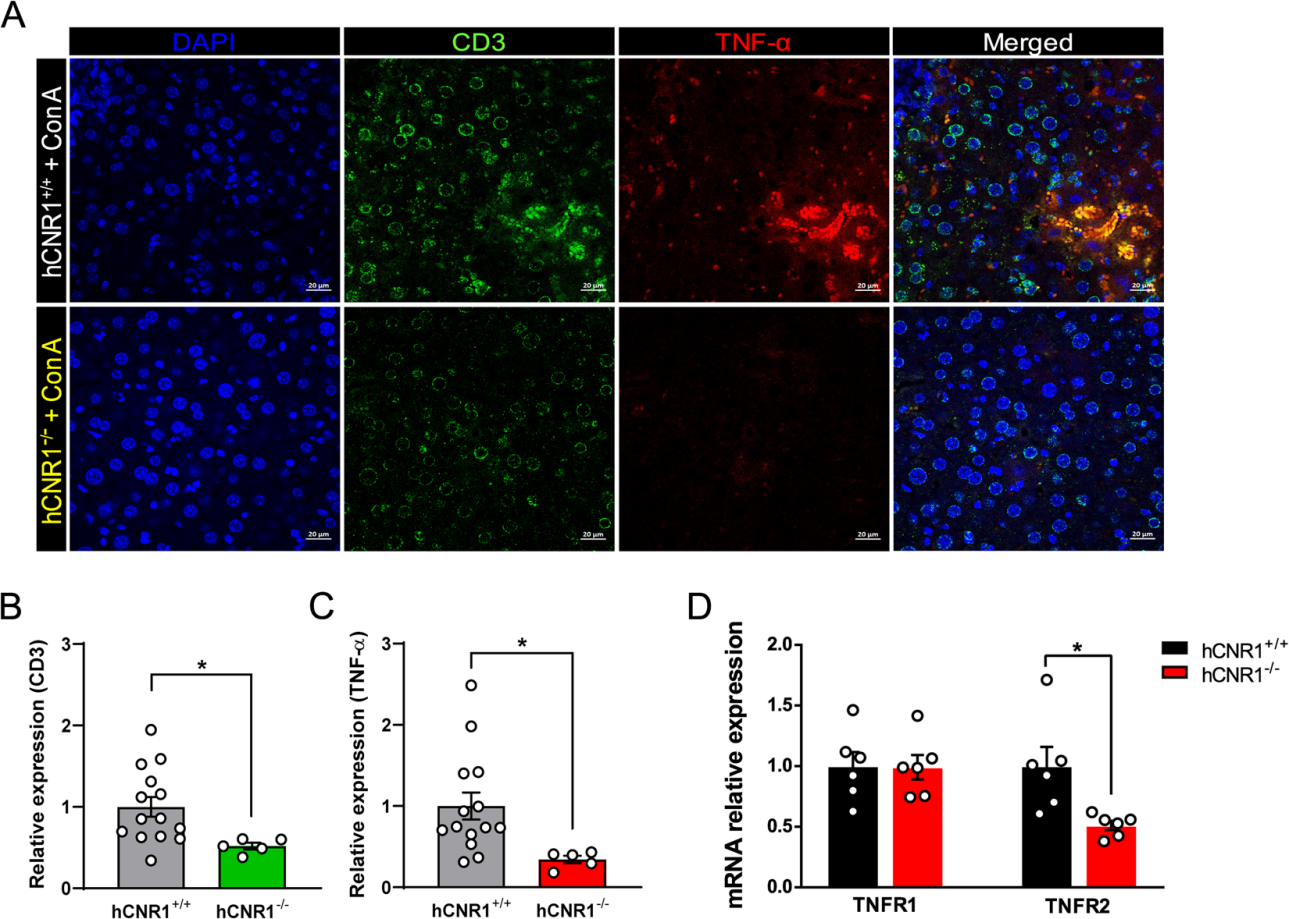
Nullification of hepatic CB1R attenuates Con A-induced liver damage via cell membrane-bound TNF-α. **A**–**C** Liver samples without permeabilization were fixed and sectioned, and immunostained for CD3, TNF-α, and DAPI (×40). Relative quantifications of immunofluorescence stained tissues (**B**, **C**). **D** TNFR1 and TNFR2 mRNA expression levels. Results were expressed as mean ± S.E.M. (*p ≤ 0.05, **p ≤ 0.01, and ***p ≤ 0.001).

It has been established that membrane-bound TNF-α, expressed on NKT cells, plays a critical role in Con A-induced liver damage^26,27^. While circulating levels of TNF-α are indeed markedly lower in hCNR1^−/−^ mice compared to hCNR1^+/+^ mice after Con A injection (Fig. 3A), it is the membrane-bound TNF-α interacting with the TNF receptor 2 (TNFR2) that leads to apoptosis^19^. We found that some infiltrating CD3-positive cells were positive for TNF-α as well, and these double-positive populations were drastically reduced in hCNR1^−/−^ mice (Fig. 5A–C) while gene expression of TNFR2, but not TNFR1, was lower in livers from hCNR1^−/−^ compared to hCNR1^+/+^ mice (Fig. 5D). These data indicate that hCNR1^−/−^ mice are resistant to Con A-induced liver damage by cell-anchored TNF-α as a ligand of TNFR2.

### Both hepatocyte-specific deletion and pharmacological inhibition of CB1R ameliorate Con A-induced hepatocyte injury

**Fig. 6:**
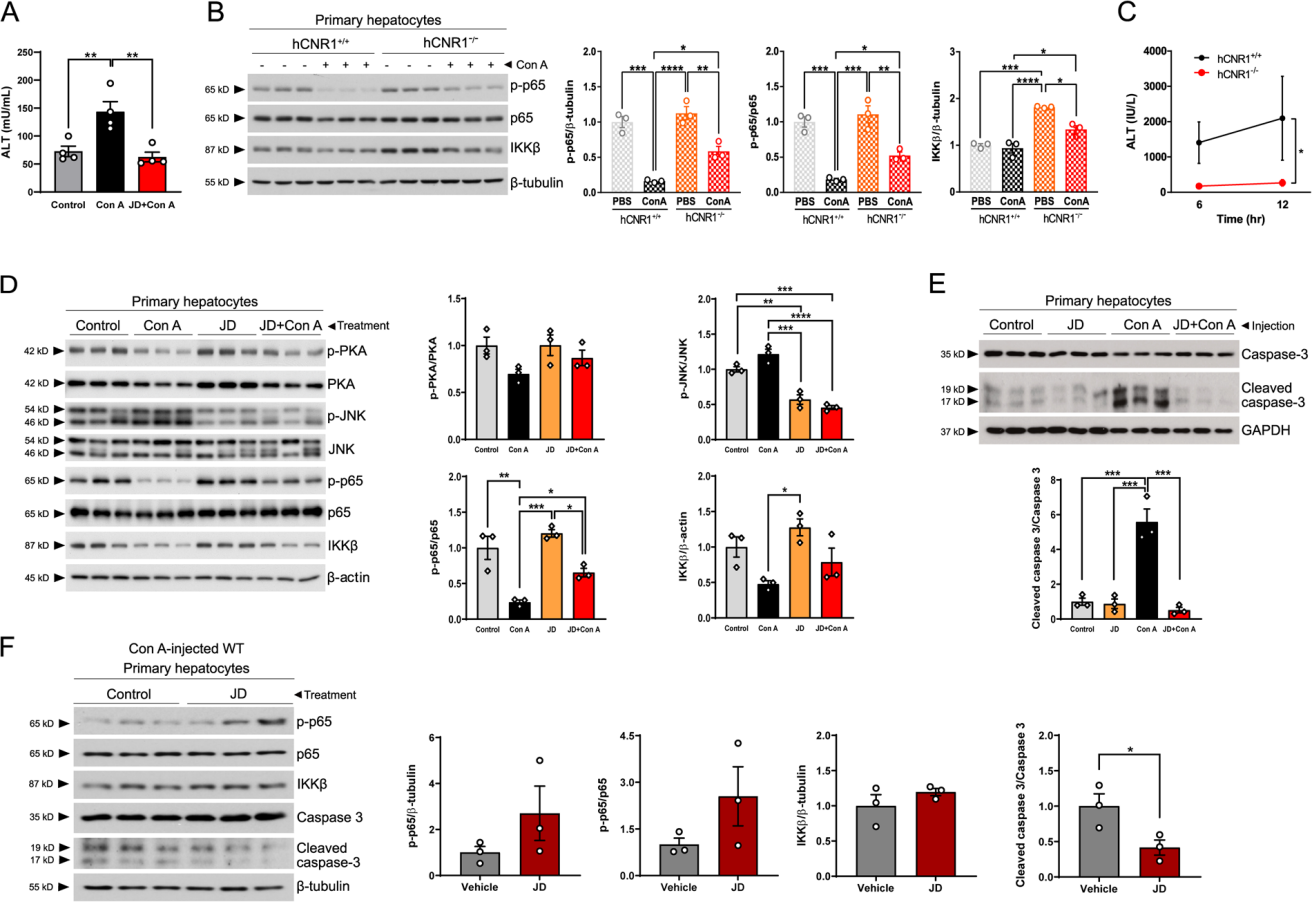
Hepatocyte-specific CB1R plays a pivotal role in response to inflammation. **A** Serum alanine aminotransferase (ALT) levels (mU/mL) in Con A-administered wild-type mice (*n* = 4 per each group). C57BL/6J background mice were injected intraperitoneally with vehicle (DMSO) or JD-5037 (3 mg/kg). After 1 h, they were given Con A (5 mg/kg) via tail vein and 6 h later blood was collected for ALT levels. **B** hCNR1^+/+^ and hCNR1^−/−^ mice were injected with vehicle (PBS) or Con A (5 mg/kg). Hepatocytes were isolated 6 h later and cultured overnight. Protein expression levels of phosphorylated and total p65 and IKKβ were measured by immunoblotting. **C** ALT levels in the medium of the hepatocytes in response to Con A treatment (50 μg/mL) after 6- and 12-h. **D** Hepatocytes were isolated from C57BL/6J mice (*n* = 4) and treated with JD-5037 (100 nM). After 4 h, PBS or Con A (50 μg/mL) were added to the media and hepatocytes incubated overnight. Proteins were extracted and protein expression levels of ratio of p-PKA/PKA, p-JNK/JNK, and p-p65/p65, and total IKKβ were quantified. **E** C57BL/6J mice were injected intraperitoneally with vehicle (DMSO) or JD-5037 (3 mg/kg). After 1 h, they were given Con A (5 mg/kg) via tail vein and hepatocytes were isolated 6 h later. Proteins were extracted from cell lysates, followed by the detection of total and cleaved caspase 3. **F** C57BL/6J male mice were administered Con A (5 mg/kg) via intravenous injection and then isolation of hepatocytes was conducted 6 h post-injection. After overnight incubation, hepatocytes were treated with vehicle (DMSO) or JD-5037 (10 nM) for 1 h, then proteins were extracted and immunoblotted for detection of phosphorylated and total p65, total IKKβ, and ratio of cleaved caspase 3/total caspase 3. Density of bands was quantified. Results were expressed as mean ± S.E.M. (*p ≤ 0.05, **p ≤ 0.01, ***p ≤ 0.001, and ^****^p ≤ 0.0001).

The above findings indicate that deletion of CB1R in liver plays a protective role in liver damage by application of Con A. To confirm whether this protection is affected by peripheral CB1R antagonism without compensatory mechanisms in response to *Cnr1* gene knockout, we injected JD-5037 (3 mg/kg), a pharmacologic inverse agonist of peripheral CB1R^28^, to mice prior to Con A (5 mg/kg). Consistent with the results in hCNR1^−/−^ mice, JD-5037 protected against liver injury as demonstrated by normal ALT levels in circulation (Fig. 6A). Although we demonstrated that hepatocyte-specific deletion of CB1R in mice protects against liver damage caused by TNF-α after Con A injection and a CB1R antagonist also protects against liver injury, we cannot rule out the possibility that hepatocytes may have been influenced by paracrine signaling from neighboring non-parenchymal cells, such as the liver-resident macrophages and Kupffer cells (KCs). In fact, it has recently been reported that knockdown of CB1R in KCs promotes an anti-inflammatory profile including a lower TNF-α expression^29^. Thus, we isolated hepatocytes to investigate the effect of the genetically or pharmacologically CB1R-nullified hepatocytes alone on acute liver damage. We injected both hCNR1^+/+^ and hCNR1^−/−^ mice with Con A (5 mg/kg), and 6 h later we isolated hepatocytes and cultured them overnight. In concordance with the in vivo data, the levels of phospho-p65 were significantly higher in isolated hepatocytes from hCNR1^−/−^mice compared to the wild-type counterpart, confirming the anti-apoptotic role of NF-κB p65 subunit (Fig. 6B). IKKβ is required for NF-kB activation and suppression of TNF-α-mediated liver apoptosis. The levels of IKKβ were increased in hepatocytes from hCNR1^−/−^mice compared to wild-type hepatocytes, explaining the increased levels of phospho-p65 (Fig. 6B), in keeping with the whole liver findings described above. Moreover, hepatocytes from hCNR1^−/−^mice had significantly less damage, as shown by the ALT levels in the medium, compared to those from hCNR1^+/+^ mice (Fig. 6C). We further examined if pharmacological inhibition of CB1R would result in similar protection from Con A. To study the effect of pharmacological CB1R inhibitor in acute liver damage, we isolated hepatocytes from C57Bl/6J mice and treated them with JD-5037 (100 nM). Four hours later, Con A (50 μg/mL) was added to the hepatocytes which were then incubated overnight. JD-5037 significantly suppressed JNK activation levels and there was a higher p-p65/p65 ratio and increased total IKKβ similar to what was observed in CB1R-deleted animal models (Fig. 6D). Additionally, we injected wild-type mice with JD-5037 (3 mg/kg). One hour later, we administered Con A (5 mg/kg) via the tail vein, and 6 h later we again isolated primary hepatocytes from the injected mice. Consistent with in vivo results from liver samples, we found that Con A treatment led to activation of caspase 3 in hepatocytes from hCNR1^+/+^ mice, whereas antagonism of CB1 receptor clearly protected against Con A-induced programed cell death (Fig. 6E). To evaluate a potential role of hepatic CB1R in amelioration of liver damage, we gave Con A (5 mg/kg) via intravenous injection to wild-type animals, and then isolated hepatocytes from liver-damaged mice after 6 h. Hepatocytes were incubated overnight and treated with either vehicle (DMSO) or JD-5037 (10 nM) for 1 h. We found that phosphorylated p65 protein expression levels were increased and there was less expression of active caspase 3 in CB1R antagonist-treated hepatocytes compared to controls (Fig. 6F).

## Discussion

Over the last two decades, the ECS has emerged as a player involved in diverse causes of liver inflammation^8,30^. Although two studies have reported that exogenous and endogenous cannabinoids attenuate autoimmune hepatitis, in contrast to our results^15,31^, the beneficial effects were upon simultaneous engagement of both CB1 and CB2 receptors. A role for CB2R on immune cells has been widely reported and it therefore has potential to play a role in Con A-induced immunological disorder, whereas CB1R is not expressed on immune cells. In the present study, we measured CB2R (*Cnr2*) gene expression levels in livers from hCNR1^+/+^ and hCNR1^−/−^ mice under both untreated and Con A-treated conditions. Although genetic compensation in response to gene knockout is a widespread phenomenon, we observed that lack of hepatic CB1R did not result in transcriptional genetic compensation of CB2R in either uncompromised or Con A-treated livers. We did not measure tissue levels of ECs such as 2-AG and anandamide in this study, one possibility is that hepatic injury and disease conditions such as hepatocellular carcinoma in humans^32^ and liver cirrhosis in rats^33^ lead to increased amounts of intra-hepatic ECs that then were permissive to hepatocyte damage in the presence of a toxin such as Con A. CB1R is constitutively active in hCNR1^+/+^ mice. However, tissue-specific genetic ablation and peripheral blockade of CB1R would have suppressed its activity^34^. Future experiments are required to more fully understand the CB1 receptor-mediated upstream events in acute liver disease.

Our studies highlight a role for CB1R in toxin-induced liver damage. To understand the broader role of *Cnr1* gene, we performed microarray analyses using hepatocytes from hCNR1^+/+^ and hCNR1^−/−^ mice. GO analysis based on differentially expressed genes revealed greater involvement of *Cnr1* gene in liver inflammation and hepatitis. We then analyzed these expression profiles to search for networks and pathways, which might explain the seemingly negative effects of CB1R on proinflammatory cytokines and hepatic apoptosis. Although we mainly focused on cAMP-associated *adcy* genes downstream from EC signaling, we additionally noted downregulation of *Ndufa4l2* and *Pik3cd* gene expression involved in liver inflammation. NADH dehydrogenase (ubiquinone) 1 alpha subcomplex 4-like 2 (NDUFA4L2) affects cell viability and mitochondrial dysfunction and is known to be downregulated in CB1R-deleted podocytes^35^. Also, phosphoinositide 3-kinase delta (PI3Kδ, *Pik3cd* gene) regulates B and T cells^36^, which is coupled to CB1R in CHO cells^37^. Further study is needed to examine for any roles of such potential genes in liver inflammation.

**Fig. 7:**
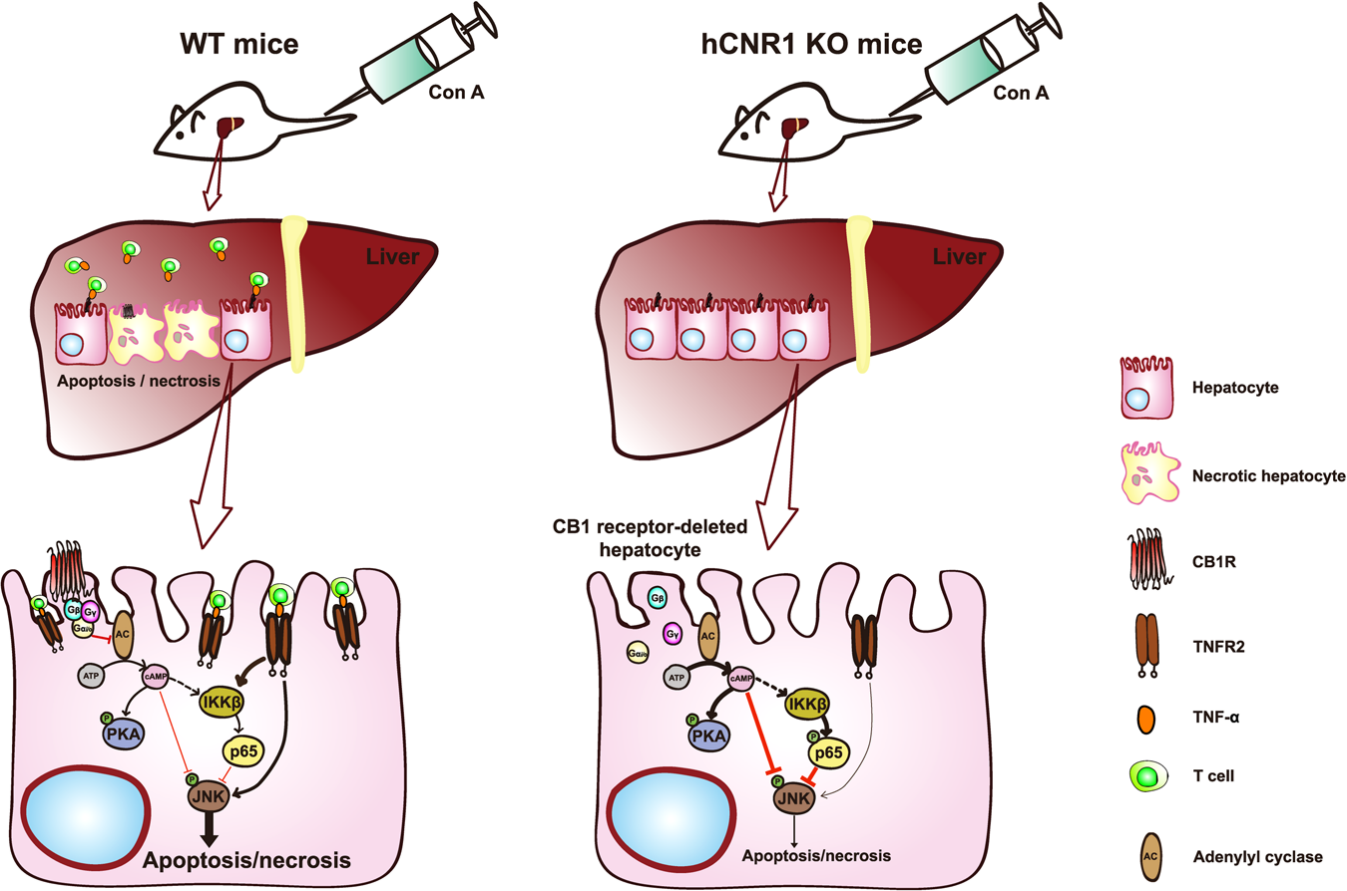
Schema of the suggested protective mechanism of hepatic cannabinoid 1 (CB1) receptor nullification/blockade on liver injury by Con A treatment. Nullification of hepatocyte-specific CB1 receptor reduces infiltration of T cells containing membrane-bound TNF-α in liver and reduces TNFR2 levels in isolated hepatocytes. Interaction between membrane-bound TNF-α and TNFR2 results in the activation of JNK and apoptotic cell death in WT mice. hCNR1^−/−^ mice also have increased cAMP levels and PKA activation, and consequently decreased phosphorylation of JNK. Additionally, CB1 receptor-deleted hepatocytes have higher expression of phosphorylated NF-κB subunit, p65 (S536), via activation of IKKβ resulting in the protection of hepatocytes against apoptotic cell death.

Con A injection causes a cytokine storm accompanied by antigen-independent T cell activation, resulting in the death of hepatocytes^3^. Here, we demonstrate that lack of CB1R in hepatocytes alone led to a marked decrease in proinflammatory cytokine levels. However, we cannot rule out the possibility that other immune cells such as KCs, hepatic resident macrophage, and inflammatory cells including infiltrating macrophages, T lymphocytes, neutrophils, and dendritic cells contribute to liver inflammation^38^. It has been recently reported that CB1R directly affects inflammation in KCs and hepatic stellate cells (HSCs)^29,39^. In our study, less proinflammatory cytokines account for less tissue damage as evidenced by reduced cleaved caspase 3 and cleaved PARP, and increased levels of Bcl-2. This anti-apoptotic effect results from either activation of NF-κB or suppression of JNK activation or more likely both, since JNK involvement is known to occur in Con A-induced liver damage^19^. With regards to the inhibition of JNK activation, this is likely due to elevated cAMP levels through cAMP response element-binding protein (CREB)-mediated downstream molecules such as cellular FLICE-inhibitory protein (c-FLIP) and mitogen-activated protein kinase phosphatase-1 (MKP-1) that inhibit JNK activation^40^ (Fig. 7).

Additional evidence that increased cAMP levels due to deletion or antagonism of CB1R contribute to alleviation of liver damage comes from the previous studies in which pharmacologic or genetic blockade of CB1R activates glucagon-like pepetide-1 receptor (GLP-1R), resulting in elevated cAMP levels^41–43^. More interestingly, a GLP-1R agonist counteracts against NAFLD through cAMP-PKA axis^44^. In our work, elevated cAMP levels may also be permissive to activation of IKKβ that, in turn, activates the p65 subunit of NF-κB and protects against Con A-induced liver apoptosis. In accordance with this, hepatocyte-specific IKKβ knockout mice have been shown to be highly sensitive to Con A-induced liver damage because of membrane-bound TNF-α activation^19^. We found that some infiltrating CD3^+^ T cells contain TNF-α on their cell surface in livers of hCNR1^+/+^ mice that would interact with TNFR2 expressed on hepatocytes and cause apoptotic cell death. Interestingly, the levels of these double-positive T cells were drastically reduced in livers of hCNR1^−/−^ mice. Moreover, we found that the expression level of TNFR2, but not TNFR1, was significantly lower in livers from hCNR1^−/−^ mice, indicating yet a second layer of protection against membrane bound TNF-α. Compared to p65-deficient mice, p65-sufficient mice have enhanced survival^16^. In line with these results, we observed a significant reduction in inflammatory cytokine production and cell death.

In summary, the current study provides evidence that peripheral CB1R nullification or blockade protects the liver from injury during an acute toxic insult. Global CB1R antagonism orchestrates multiple downstream signaling resulting in protection against liver injury of an insidious nature such as FLD. However, there is strong epidemiological evidence that CB1R inhibition over a long-term timeframe puts people at risk for adverse psychiatric effects and therefore CB1R antagonists need to be devoid of central psychiatric effects in order to be a useful therapeutic strategy in the pathogenesis of toxin-induced liver diseases. The present set of investigations now provides strong evidence for protection against acute toxin-induced injury by CB1R blockade in both whole liver in vivo in animals and in vitro in hepatocytes, and under such circumstances CB1R antagonism would be an acute treatment, thereby rendering any CNS effects mute. Taken together, our data define a role for CR1R antagonism in protection against liver damage by extrinsic stimuli, pointing toward the benefits of developing a therapeutic strategy involving a CB1R antagonist in the setting of a toxin-induced liver injury, especially during the acute phase of toxicity.

## Materials and methods

### Animals

All procedures for the use of the mice followed protocols approved by the National Institute on Aging (NIA) which is fully accredited by the American Association for Accreditation of Laboratory Animal Care, and all procedures were approved by the Animal Care and Use Committee of the NIA Intramural Research Program. All animals were housed at NIA. Hepatocyte-specific CB1^−/−^ mice (hCNR1^−/−^) and control animal (hCNR1^*+/+*^) littermates were developed by breeding with mice carrying the albumin-Cre gene (Alb-Cre; Jackson Laboratories, Bar Harbor, ME) to CNR1^flox/flox^ mice^22^. Male age-matched (8–14 weeks) hCNR1^−/−^ and hCNR1^*+/+*^ mice were randomly divided into two groups (*n* = 6 per group) and were given a single intravenous injection of Con A dissolved in PBS into the tail vein (Sigma-Aldrich, St. Louis, MO) at 2.0, 3.75, 5.0, and 7.5 mg/kg body weight. The concentrations of Con A used in this study were determined from previous studies^45^.

### Serum analysis

Mice were anesthetized, blood was collected by cardiac puncture, and serum was separated by centrifugation at 3000 r.p.m. for 20 min. Serum levels of ALT activity were measured with ALT assay kit (Pointe Scientific, Canton, MI) or ALT/SGPT activity assay kit (BioVision, Milpitas, CA) according to the manufacturer’s instructions.

### Cytokines assay

Cytokine expression in serum was assessed by Bio-Rad BioPlex 200 instrument equipped with Bio-Plex Manager software version 6.0 (Bio-Rad Laboratories, Hercules, CA). The levels of interleukin-6 (IL-6), interleukin-1beta (IL-1β), interleukin-17 (IL-17), interferon gamma (IFN-γ), and TNF-α were detected using the Bio-Plex Pro Mouse Cytokine Th17 Panel A 6-Plex Group l Kit (Bio-Rad Laboratories,) on a Luminex 200 system (Bio-Rad Laboratories) according to the manufacturer’s instructions.

### Histopathology

For histological processing, liver tissues were removed from each mouse and fixed in fresh 4% paraformaldehyde for 24 h. Fixed liver tissues were dehydrated using a graded series of alcohol and embedded in paraffin. The sample liver tissues were then cut into 5-µm-thick sections. The paraffin sections were de-paraffinized and hydrated. Sections were stained with hematoxylin and eosin (H&E). Imaging was conducted at ×10 and ×40 using an inverted Keyence BZ-710 microscope (King of Prussia, PA). The average area of inflammatory cell infiltration and necrosis area were assessed using HALO image analysis software, v.2.21870.31 (Indica Labs, Corrales, NM).

### cAMP assay

Livers were ground and lysed in 0.1 M HCl for determination of cAMP by ELISA. cAMP levels were determined using a cAMP ELISA kit from Enzo Life Sciences (Farmingdale, NY) according to the manufacturer’s instructions. The final concentration of cAMP was normalized to protein concentration using a BCA protein assay (Pierce Biotechnology, Rockford, IL).

### Immunoblotting

Western blot analyses was performed as previously described^46^. In brief, liver tissues were lysed in tissue lysis buffer that contained 25 mM Tris (pH 7.4), 2 mM Na_3_VO_4_, 10 mM NaF, 10 mM Na_4_P_2_O_7_, 1 mM EGTA, 1 mM EDTA, and 1% NP-40 with protease and phosphatase inhibitor cocktails. The concentrations of protein from each sample were measured by BCA protein assay (Thermoscientific, Rockford, IL). For nuclear and cytoplasmic fractionation, we used NE-PER Nuclear and Cytoplasmic Extraction Reagents (Thermoscientific). For western blotting, protein lysates were mixed with 5× Laemmli buffer and then boiled at 100 °C for 5 min. After resolving in SDS-PAGE, the proteins were transferred onto polyvinylidene fluoride (PVDF) membrane. The membrane was blocked in blocking reagent (LI-COR, Lincoln, NE) assay system for 1 h at room temperature (RT), followed by incubation with primary antibody in blocking reagent with 0.1% Tween-20 at 4 °C overnight. After probing with the specific antibody, the membrane was washed three times in 0.1% TBST, followed by incubation with secondary antibody in blocking reagent with 0.1% Tween-20 at RT for 1 h, and washed again in TBST (three times for 20 min). Anti-caspase 3, cleaved caspase 3, PARP, cleaved PARP, Bcl-2, β-tubulin, p-p65, p65, IKKβ, lamin A/C, GAPDH, p-PKA, PKA, p-JNK, JNK, p-ERK1/2, and ERK1/2 were purchased from Cell Signaling Technology (Waltham, MA) and anti-AC was obtained from Santa Cruz Biotechnology (Dallas, TX). Immunoblots were developed using a chemiluminescence assay system, and bands were visualized using X-ray films. Densitometry was performed with ImageJ (NIH) for quantifications.

### Quantitative real-time PCR

RNA was extracted using a RNeasy Mini kit (Qiagen, Valencia, CA). Purified RNA was converted into cDNA using qScript cDNA Supermix (Quanta Biosciences, Gaithersburg, MD) for mouse livers and primary hepatocytes. CB1 receptor gene expression was quantified using SYBR green (Applied Biosystems, Foster City, CA) on StepOnePlus Real-Time PCR System (Applied Biosystems) and values were normalized to 18S. The mouse primers for CB1 receptor were forward: 5′-AAGTCGATCTTAGACGGCCTT-3′ and reverse: 5′-TCCTAATTTGGATGCCATGTCTC-3′ (Integrated DNA Technologies, Coralville, IA). For CB2 receptor expression study, the exon-specific primers and fluorescent FAM-labeled and minor grove binder (MGB) conjugated probe of mCB2 genes were designed (MGB TaqMan probe: TGGGCCCAGTCTT, forward: 5′-GCCACCCAGCAAACATCTCT-3′ and reverse: 5′-GATGGGCTTTGGCTTCTTCTAC-3′) using Primer Express (Applied Biosystems, Foster City, CA, USA). Universal 18S primers were purchased from ThermoFisher Scientific. Applied Biosystems TaqMan probe (Mm01212171_s1), TaqMan™ PreAmp Master Mix (Cat: 4391128), and Fast Advanced Master Mix (Cat: 4444557) were used to detect very low level of hepatocyte CB1R. We used ten cycles for preamplification and 40 cycles for TaqMan assay using 3 µL of pre-amplificated template according to the manufacture’s protocol.

### Immunofluorescent staining of livers and quantification

Slides were rehydrated with graded series of ethanol concentrations, washed in reverse osmosis water, and then washed three times (2 min) in TBS. Antigen retrieval was performed by placing the slides in 10 mM of sodium citrate buffer (pH, 6.0; Vector Laboratories, Burlingame, CA) at 95 °C for 30 min. The slides were allowed to cool at room temperature in the citrate buffer for a further 30 min and were then rinsed in TBS as before. Sections were incubated with normal goat serum block consisting of 2% goat serum, 1% OmniPur® BSA Fraction V, 0.1% gelatin, 0.05% Tween 20, and 0.05% sodium azide (all from Sigma-Aldrich) in TBS for 1 h at room temperature. Sections were then incubated in primary antibody diluted in the same normal goat serum block, overnight at 4 °C. The following primary antibodies were used: mouse anti-TNF-alpha (1:100, Abcam, ab1793) and rat anti-CD3 (1:100, R&D Systems MAB4841). Sections were then washed three times (2 min) in 0.1% Tween-20 in 1× TBS (pH, 7.4), followed by incubation with the specific secondary antibody diluted in the normal goat serum block for 1 h at room temperature. Secondary antibodies used were goat anti-mouse IgG_1_ Alexa Fluor 568, and goat-rat Alexa Fluor 488, all from Thermo Fisher Scientific. After washing in 0.1% Tween-20 in 1× TBS as before, sections were incubated for 15 min with 4′,6-diamidino-2-phenylindole (Sigma Aldrich) for nuclear staining. Slides were next washed in TBS twice for 2 min each time before being mounted with Permafluor Mountant (Thermo Fisher Scientific). The correlation of the expression levels of CD3 and TNF-alpha in liver sections was assessed by relative quantification of respective immunofluorescence staining for each protein. Using ImageJ, the Integrated Density Value (IDV) for each channel was measured (Blue = DAPI; Green = CD3; Red = TNF-alpha). An average number of nuclei in each section was estimated by dividing the total IDV of blue channel by the average IDV of 20 selected nuclei. IDV for each protein was calculated by dividing total IDV of each respective channel by the estimated number of nuclei in each section. Relative expression levels were calculated normalizing the IDV by the average IDVs of the control group (wild-type animals) in each channel. Unpaired *t*-tests were used to check for statistical significance.

### Microarray analysis

Microarray experiments and analysis were performed as we previously described^47^. Data analysis were performed using R Studio 1.2.

### Statistical analysis

The time-course and dose-response of ALT levels were analyzed using two-way ANOVA. Quantitative data are represented as the mean ± S.E.M. Quantification analysis for western blot band density and imaging pixels and levels of cytokines was conducted using unpaired two-tailed *t* test after outlier test (*α* = 0.05). GraphPad Prism (Prism 7; GraphPad Inc.) was used to perform statistical analysis; *p ≤ 0.05, **p ≤ 0.01, ***p ≤ 0.001, and ****p ≤ 0.0001 were considered statistically significant (* compared to control).

## Supplementary information

Supplementary figures

Supplementary Figure Legends

## Data Availability

The microarray data have been deposited in the Gene Expression Omnibus database under accession code GSE148309.
