# Supplementary figures and images for: Hepatocyte cannabinoid 1 receptor nullification alleviates toxin-induced liver damage via NF-κB signaling
Source: Cell Death Dis. 2020 Dec 9;11(12):1044. doi: 10.1038/s41419-020-03261-8 (PMC7726564; doi:10.1038/s41419-020-03261-8)

A

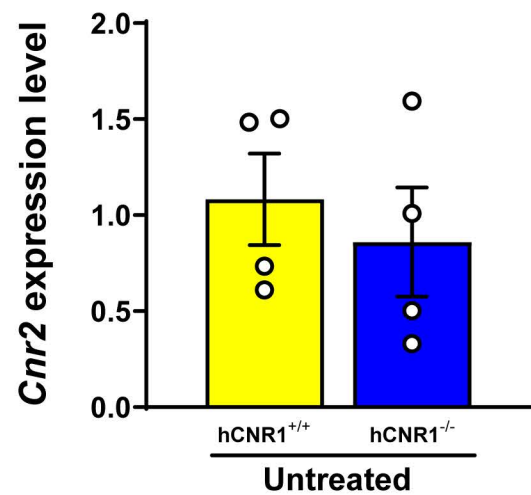

B

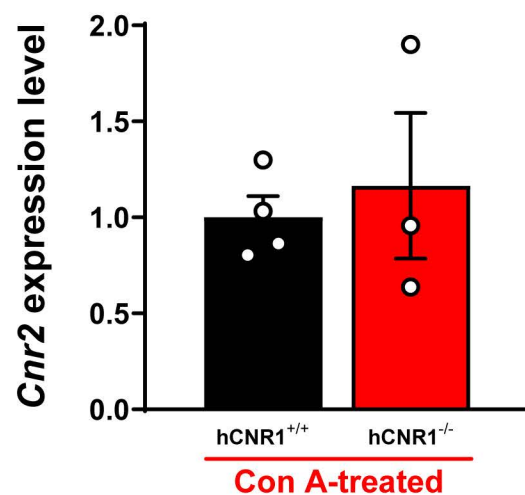

A

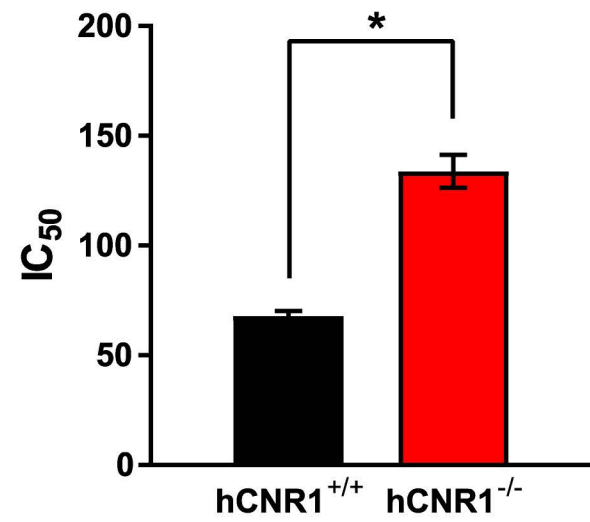

B

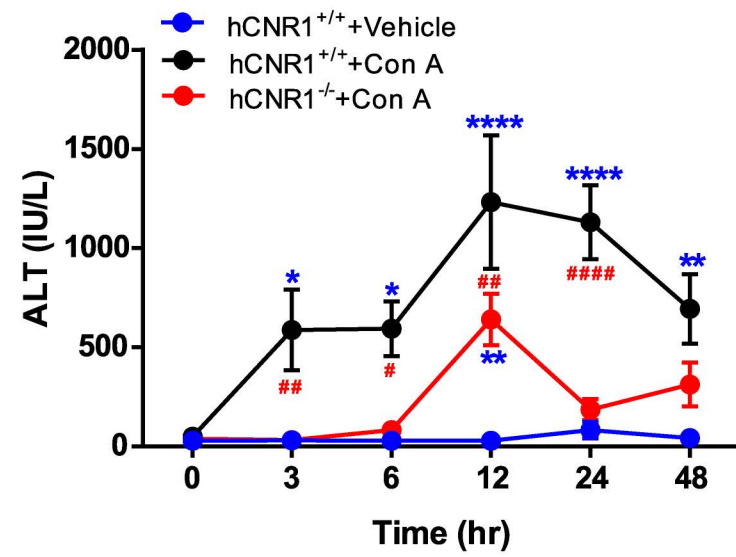

C

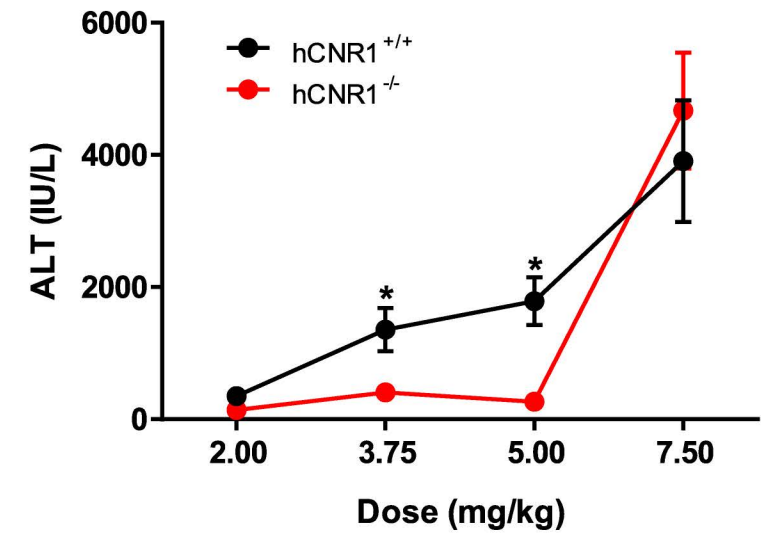

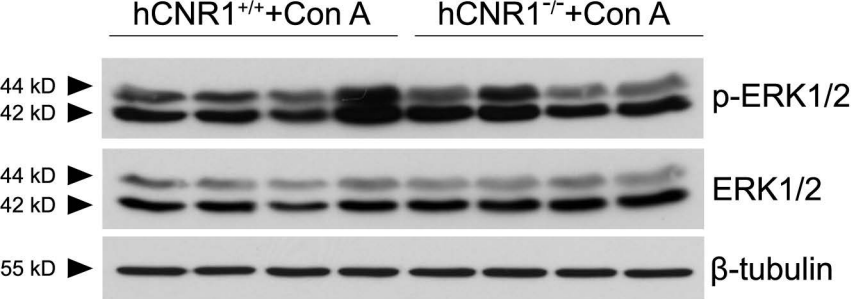

Supplement: Supplementary file 1 — Supplementary figures [file 41419_2020_3261_MOESM1_ESM.pdf]
