# Supplementary material for: Hepatocyte cannabinoid 1 receptor nullification alleviates toxin-induced liver damage via NF-κB signaling
Source: Cell Death Dis. 2020 Dec 9;11(12):1044. doi: 10.1038/s41419-020-03261-8 (PMC7726564; doi:10.1038/s41419-020-03261-8)
Supplement: Supplementary file 2 — Supplementary Figure Legends [file 41419_2020_3261_MOESM2_ESM.pdf]

Supplementary data figure legends

Supplementary Fig. 1. Hepatic *Cnr2* expression levels, Con A-treated and untreated conditions. Gene expression using RNA extracted from livers of $hCNR1^{+/+}$ and $hCNR1^{-/-}$ mice, untreated (n=4, Fig. S1A) and Con A-treated (5 mg/kg, n=3-4, Fig. S1B).

Supplementary Fig. 2. (A) Hepatocytes were isolated from $hCNR1^{+/+}$ and $hCNR1^{-/-}$ mice (n=2). After overnight incubation hepatocytes were subjected to treatment of various concentrations of Con A dissolved in PBS. Cell viability was measured using MTT (0.5 mg/ml) assay for 4 hours incubation at 37°C. (B-C) Plasma alanine aminotransferase (ALT) levels in Con A-administrated $hCNR1^{+/+}$ and $hCNR1^{-/-}$ mice in time-course with 5 mg/kg (n=4-5) and dose-response (n=3-5) curve at 24 hours post injection. Results were expressed as mean ±S.E.M. ($^{*}p \leq 0.05$, $^{**}p \leq 0.01$, $^{****}p \leq 0.0001$ and $^{\#}p \leq 0.05$, $^{\#\#}p \leq 0.01$, $^{\#\#\#\#}p \leq 0.0001$)

Supplementary Fig. 3. $hCNR1^{+/+}$ and $hCNR1^{-/-}$ mice were injected Con A (5.0 mg/kg) for 12 hours (n=4). Protein extracts from liver homogenates were immunoblotted against anti-phospho-extracellular signal-regulated kinase 1/2 (ERK 1/2) and total ERK 1/2 and detected by autoradiographic signals.
